# Impact of cystatin C-derived glomerular filtration rate in patients undergoing transcatheter aortic valve implantation

**DOI:** 10.3389/fcvm.2023.1035736

**Published:** 2023-04-21

**Authors:** Yusuke Kure, Tsukasa Okai, Yasuhiro Izumiya, Hisako Yoshida, Kazuki Mizutani, Tomohiro Yamaguchi, Mana Ogawa, Atsushi Shibata, Asahiro Ito, Yosuke Takahashi, Toshihiko Shibata, Daiju Fukuda

**Affiliations:** ^1^ Department of Cardiovascular Medicine, Osaka Metropolitan University Graduate School of Medicine, Osaka, Japan; ^2^ Department of Medical Statistics, Osaka Metropolitan University Graduate School of Medicine, Osaka, Japan; ^3^ Division of Cardiology, Department of Medicine, Kindai University Faculty of Medicine, Osaka, Japan; ^4^ Department of Cardiovascular Surgery, Osaka Metropolitan University Graduate School of Medicine, Osaka, Japan

**Keywords:** TAVI, aortic stenosis, cystatin c, CKD, creatinine

## Abstract

**Background:**

Chronic kidney disease (CKD) impacts prognosis in patients undergoing transcatheter aortic valve implantation (TAVI). While estimated glomerular filtration rate (eGFR) calculated from serum creatinine [eGFR (creatinine)] is affected by body muscle mass which reflects frailty, eGFR calculated from serum cystatin C [eGFR (cystatin C)] is independent of body composition, resulting in better renal function assessment.

**Methods:**

This study included 390 consecutive patients with symptomatic severe aortic stenosis (AS) who underwent TAVI, and measured cystatin C-based eGFR at discharge. Patients were divided into two groups, with or without CKD estimated with eGFR (cystatin C). The primary endpoint of this study was the 3-year all-cause mortality after TAVI.

**Results:**

The median patient age was 84 years, and 32.8% patients were men. Multivariate Cox regression analysis indicated that eGFR (cystatin C), diabetes mellitus, and liver disease were independently associated with 3-year all-cause mortality. In the receiver-operating characteristic (ROC) curve, the predictive value of eGFR (cystatin C) was significantly higher than that of eGFR (creatinine). Furthermore, Kaplan–Meier estimates revealed that 3-year all-cause mortality was higher in the CKD (cystatin C) group than that in the non-CKD (cystatin C) group with log-rank *p* = 0.009. In contrast, there was no significant difference between the CKD (creatinine) and non-CKD (creatinine) groups with log-rank *p* = 0.94.

**Conclusions:**

eGFR (cystatin C) was associated with 3-year all-cause mortality in patients who underwent TAVI, and it was superior to eGFR (creatinine) as a prognostic biomarker.

## 1. Introduction

Aortic stenosis (AS) frequently causes left ventricular outflow impairment and is a common public health problem in an aging society (1, 2). Transcatheter aortic valve implantation (TAVI) has demonstrated comparable outcomes with surgical aortic valve replacement, and is the preferred treatment option for AS patients from all surgical risk categories considered for a bioprosthetic valve (3–6). Although clinical outcomes after TAVI are generally good, there are some patients at a higher risk of short- and long-term mortality and morbidity. The remnant problem in treating AS is to investigate the relationship between potential risk to the patients and their long-term prognosis.

One of the prognostic factors impacting patients who undergo TAVI is CKD (7–9). Although the global index of renal function is eGFR (creatinine), serum creatinine can be affected by muscle mass and dietary protein intake, which decreases with increasing age (10). However, serum cystatin C level is another marker of renal function that is considered potentially superior to serum creatinine level for estimating renal function because it is produced constantly by most nucleated cells (11). Moreover, cystatin C production has been reported to be unaffected by age, gender, or muscle mass. Thus, renal function can be assessed more accurately using eGFR (cystatin C) than eGFR (creatinine) (12–15). However, the prognostic value of eGFR (cystatin C) has not been explored in patients who underwent TAVI. Therefore, this study aimed to evaluate the 3-year prognostic impact of CKD calculated from cystatin C after TAVI.

## 2. Methods

### 2.1. Study population

**Figure 1:**
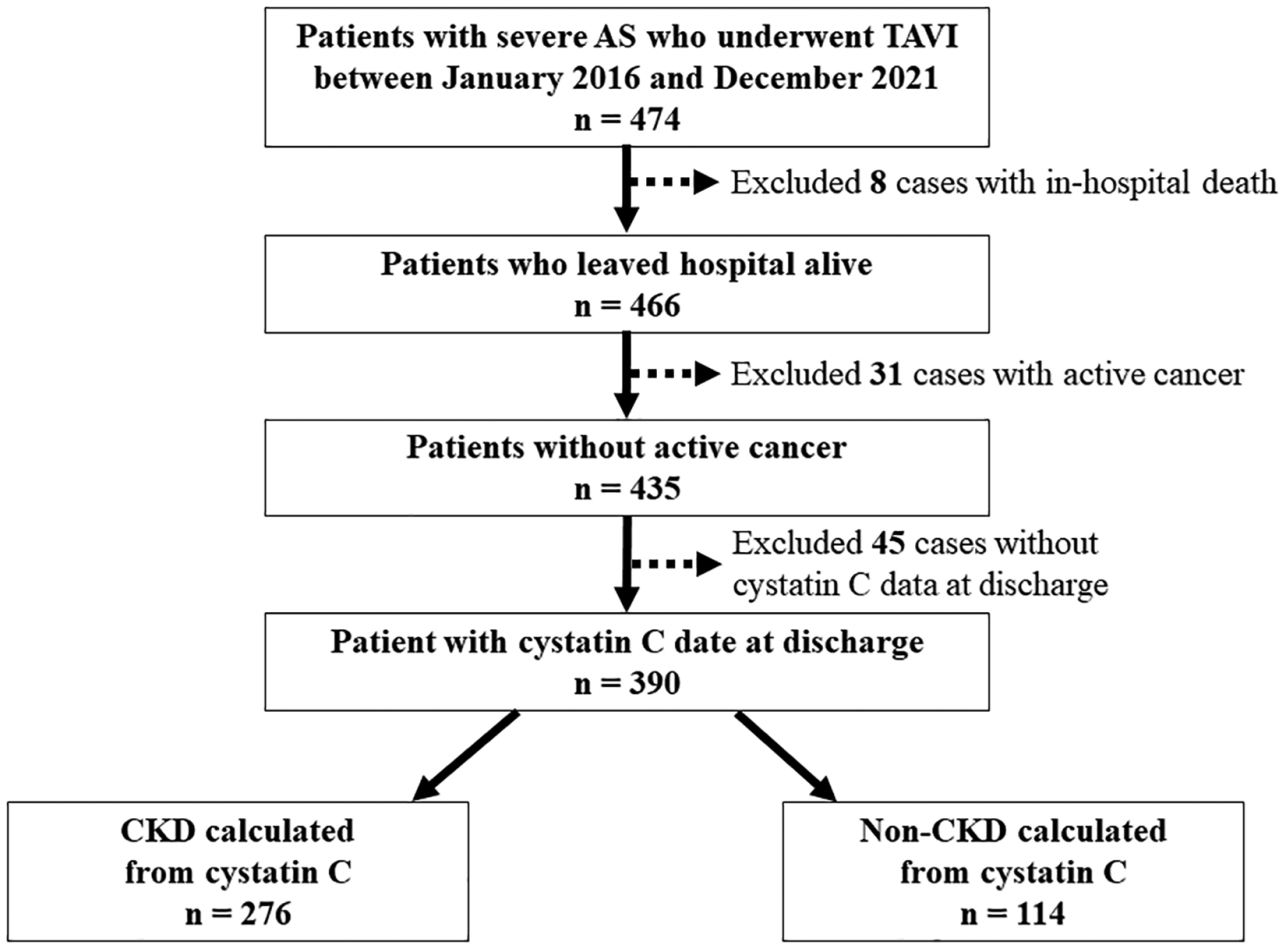
Flowchart of patient selection. AS, aortic stenosis; CKD, chronic kidney disease; TAVI, transcatheter aortic valve implantation.

This single-center prospective observational study included 474 consecutive patients with symptomatic severe AS who underwent TAVI at Osaka Metropolitan University Hospital between January 2016 and December 2021 (Figure 1). The inclusion criteria were presence of symptomatic and degenerative AS, mean aortic valve pressure gradient (mAVPG) > 40 mmHg or jet velocity > 4.0 m/s, or aortic valve area (AVA) 1.0 cm^2^ (or aortic valve area index < 0.6 cm^2^/m^2^), according to the guidelines for valvular heart disease by the European Society of Cardiology and the European Association for Cardio-Thoracic Surgery (16). The indications for TAVI were determined based on the clinical consensus of a multidisciplinary team, including cardiac surgeons, interventional cardiologists, anesthesiologists, and imaging specialists. We excluded patients who died in the hospital due to peri-procedural complications. In addition, we excluded patients with active cancer because cancer may be an independent risk factor for death and patients without serum cystatin C data at the time of discharge. The protocol of this study was in accordance with the guidelines of the Declaration of Helsinki and was approved by our institutional ethics committee (approval number: 2021-064). All patients gave informed written consent for participating in the study.

### 2.2. TAVI procedure

We selected transfemoral approach as our first option and an alternative access (transapical, transaortic, transsubclavian) for patients with excessively narrow access routes or aortic arch atheroma. We performed TAVI in a hybrid operating room under general anesthesia, except for eight patients who underwent conscious sedation because of pulmonary dysfunction. Transcatheter heart valves were used either balloon-expandable (Edwards Sapien XT or Sapien 3 Transcatheter Heart Valve; Edwards Lifesciences, Irvine, CA, USA) or self-expandable (Medtronic classic CoreValve or CoreValve Evolut R/Pro/Pro+; Medtronic, Inc., Minneapolis, MN, USA). We chose Balloon-expandable valves as the first option, while self-expandable valves were reserved for patients with a narrow aortic annulus.

### 2.3. Data collection

All data were collected prospectively from patient records. Pre-procedural enhanced multi-slice computed tomography data were obtained to evaluate the annulus area, perimeter of the annulus, and diameter of the ST junction. The data were measured using the SYNAPSE VINCENT (Fujifilm, CO., Ltd, Japan). Blood test screens including serum creatinine and serum cystatin C were performed upon admission. The follow-up protocol in this study includes at discharge, 1 month, 3 months, 6 months, and every 6 months thereafter following TAVI. Patients who did not attend the regular follow-up visits were contacted by phone to confirm their survival. We measured serum creatinine and cystatin C in all patients at discharge and calculated eGFR (creatinine) and eGFR (cystatin C). The formula for calculating the eGFR is as follows: eGFR (creatinine) = 194 × Serum creatinine (mg/dl)^−1.094^ × Age (year)^−0.287^ × 0.739 (if female), eGFR (cystatin C) = 104 × Serum cystatin C (mg/dl) × 0.996^Age (year) ^× 0.929 (if female) −8 (17, 18). CKD was defined as eGFR < 60 ml/min/1.73 m^2^, which was estimated from both serum creatinine and cystatin C (19). We divided the study population into two groups (CKD or non-CKD) calculated from serum creatinine and cystatin C, respectively. Other complications during TAVI, including the procedural, were evaluated according to the Valve Academic Research Consortium-3 criteria (20).

### 2.4. Study endpoint

The primary endpoint of this study was 3-year all-cause mortality after TAVI.

### 2.5. Statistical analysis

Categorical variables were expressed as summarized using means of counts and percentages, and continuous variables were expressed as summarized using medians and interquartile ranges (quartiles 1 to 3).Continuous and categorical variables between the two groups were compared with the Wilcoxon rank sum and chi-square tests, respectively. We evaluated the impact of eGFR (creatinine) and eGFR (cystatin C) on the endpoint using univariable and multivariable Cox regression analyses with 95% CI. To avoid the issue of multicollinearity, we used four models, one including eGFR (cystatin C) and the other including eGFR (creatinine). This multivariate model was built by selecting variables that satisfied the entry criterion of *p* < 0.05 and a 95% CI that exceeded 1 in the univariate analysis. The other two were multivariate models including eGFR (cystatin C) or eGFR (creatinine) and age and male sex. Three-year all-cause mortality was estimated using the Kaplan–Meier method, and the difference between the two groups (CKD calculated from serum creatinine and cystatin C, respectively) was evaluated using the log-rank test. The validity of the eGFR (creatinine) and eGFR (cystatin C) for estimating the 3-year all-cause mortality was evaluated using receiver-operating characteristic (ROC) curves, and area under the curve (AUC) of the eGFR (creatinine) and eGFR (cystatin C) were assessed using an ROC analysis tool based on DeLong's method (21). The statistical analyses were performed using the R software package (version 4.2.0; R Development Core Team, Vienna, Austria). The significance level of a statistical hypothesis testing was set at 0.05 and that of the alternative hypothesis was two-sided.

## 3. Results

### 3.1. Baseline patient characteristics, peri- and post-procedural findings

Among 474 possible TAVI candidates, we excluded 8 patients who died in the hospital and 31 patients with active cancer and 45 patients without serum cystatin C data at the time of discharge (Figure 1). Baseline patient characteristics are listed in Table 1. The median patient age was 84 years (interquartile range, 81–88 years), and 32.8% patients were men. The median society of thoracic surgeons (STS) risk and Clinical Frailty Scale scores were 6.46% (4.63–9.29%) and 4 (3–5), respectively. The median eGFR (cystatin C), eGFR (creatinine), and brain natriuretic peptide (BNP) on admission were 49.0 (36.1–60.1), 49.3 (39.5–63.5), and 180.3 (76.7–434.1), respectively. Evaluation of preoperative transthoracic echocardiograms showed that the median left ventricular ejection fraction (LVEF) was 61% (55%–65%) and the median aortic valve area with Doppler method was 0.66 cm²/m² (0.57–0.73 cm²/m²), with a mAVPG of 45 mmHg (36–60 mmHg).

**Table 1:** Baseline clinical characteristic of study patients.

Baseline Clinical Characteristic	Total *n* = 390	CKD calculated from cystatin C *n* = 276	Non-CKD calculated from cystatin C *n* = 114	*p*-value
Age, years	84 (81–88)	85 (82–88)	83 (80–87)	<0.001
Male sex, *n* (%)	127 (32.8)	85 (30.8)	43 (37.7)	0.19
BSA, m^2^	1.43 (1.31–1.55)	1.42 (1.30–1.54)	1.47 (1.33–1.59)	0.08
NYHA Class III or IV, *n* (%)	85 (21.8)	71 (25.7)	14 (12.3)	0.003
STS score	6.46 (4.63–9.29)	7.16 (5.04–10.22)	5.42 (3.64–6.98)	<0.001
CFS	4 (3–5)	4 (3–5)	4 (3–4)	0.07
**Comorbidity, *n* (%)**				
Diabetes mellitus	100 (25.6)	70 (25.4)	30 (24.6)	0.90
Hypertension	348 (89.2)	248 (89.9)	100 (87.7)	0.59
Dyslipidemia	222 (56.9)	154 (55.8)	68 (59.6)	0.50
Coronary artery disease	110 (28.2)	80 (29.0)	30 (26.3)	0.62
Peripheral artery disease	65 (16.7)	55 (19.9)	10 (8.8)	0.006
Atrial fibrillation	67 (18.5)	60 (21.7)	12 (10.5)	0.009
Previous stroke	47 (12.1)	37 (13.4)	10 (8.8)	0.23
Liver disease	16 (4.1)	14 (5.1)	2 (1.8)	0.17
Pulmonary disease	36 (9.2)	31 (11.2)	5 (4.4)	0.03
**Preprocedural laboratory data**				
Albumin, g/dl	3.8 (3.5–4.1)	3.7 (3.4–4.0)	4.0 (3.7–4.2)	<0.001
eGFR from cystatin C, ml/min/1.73 m²	49.0 (36.1–60.1)	40.0 (31.5–49.6)	68.1 (58.5–76.8)	<0.001
eGFR from creatinine, ml/min/1.73 m²	49.3 (39.5–63.5)	44.0 (35.8–53.0)	66.4 (56.9–75.0)	<0.001
Natrium, mEq/L	140 (138–142)	141 (138–142)	140 (139–141)	0.03
Hemoglobin, g/dl	11.5 (10.3–12.6)	11.1 (10.0–12.4)	12.2 (10.8–13.0)	<0.001
BNP, pg/ml	180.3 (76.7–434.1)	234.1 (105.9–496.0)	99.9 (49.6–195.5)	<0.001
**Preprocedural echocardiographic data**				
LVEF, %	61 (55–65)	60 (54–65)	63 (60–65)	0.03
Peak AV velocity, m/s	4.5 (4.1–5.1)	4.4 (4.1–5.1)	4.6 (4.1–5.0)	0.63
Mean AVPG, mmHg	45 (36–60)	44 (35–61)	46 (39–59)	0.49
AVA, cm²	0.66 (0.57–0.73)	0.65 (0.57–0.73)	0.66 (0.59–0.73)	0.53
Moderate or severe AR, *n* (%)	36 (9.2)	25 (9.1)	11 (9.6)	0.85
Moderate or severe MR, *n* (%)	44 (11.3)	35 (12.7)	9 (7.9)	0.22
**Preprocedural CT data**				
Annulus area, mm²	393 (342–443)	392 (342–448)	394 (345–438)	0.78
Perimeter, mm	70.6 (66.1–75.3)	70.5 (66.0–75.6)	70.7 (66.5–74.4)	0.98

Categorical variables are shown as numbers (percentages) and continuous variables are shown as medians (25–75th percentiles).

BSA, body surface area; NYHA, New York heart association; STS, society of thoracic surgeons *p*redictive risk of mortality; CFS, clinical frailty scale; eGFR, estimated glomerular filtration rate; BNP, brain natriuretic peptide; LVEF, left ventricle ejection fraction by modified simpson methods; AV, aortic valve; AVPG, aortic valve pressure gradient; AVA, aortic valve area; AR, aortic regurgitation; MR, mitral regurgitation; CT, computed tomography.

Table 2 displays information about the peri- and post-procedural outcomes. Among the total study population, 91.0% patients underwent transfemoral TAVI, while 67.9% underwent balloon-expandable TAVI. Peri-procedural complications included permanent pacemaker implantation, disabling stroke, acute kidney injury, and bleeding in 4.1%, 2.8%, 4.1% and 8.5% patients, respectively. Echocardiography revealed that post-procedural mAVPG and EOA were 9 mmHg and 1.56 cm²/m², respectively.

**Table 2:** Peri– and postprocedural outcome information.

Procedural and Outcome Information	Total *n* = 390	CKD calculated from cystatin C *n* = 276	Non-CKD calculated from cystatin C *n* = 114	*p*-value
Transfemoral, *n* (%)	355 (91.0)	250 (90.6)	105 (92.6)	0.70
SAPIEN XT, *n* (%)	18 (4.6)	13 (4.7)	5 (4.4)	1.0
SAPIEN 3, *n* (%)	247 (63.3)	175 (63.4)	72 (63.2)	1.0
Core valve, *n* (%)	3 (0.8)	3 (1.1)	0 (0.0)	0.56
Evolut R, *n* (%)	41 (10.5)	30 (10.9)	11 (9.6)	0.86
Evolut Pro/ Pro+, *n* (%)	81 (20.8)	55 (19.9)	26 (22.8)	0.58
Valve size, mm	26 (23–26)	26 (23–26)	26 (23–26)	0.74
**Periprocedural variable**				
Procedual time, min	55 (40–80)	60 (45–86)	50 (40–70)	0.004
Local anesthesia, *n* (%)	8 (2.1)	7 (2.5)	1 (0.9)	0.45
Contrast, ml	62 (54–76)	60 (53–73)	65 (54–78)	0.06
**Periprocedural Complications, *n* (%)**				
Coronary obstruction	8 (2.3)	6 (2.2)	3 (2.6)	0.72
Permanent pacemaker implantation	16 (4.1)	12 (4.3)	4 (3.5)	1.0
Disabling stroke	11 (2.8)	7 (2.5)	4 (3.5)	0.74
Acute kidney injury	16 (4.1)	16 (5.8)	0 (0.0)	0.004
All bleeding	33 (8.5)	28 (10.1)	5 (4.4)	0.07
Life–threatening/Major bleeding	18 (4.6)	17 (6.2)	1 (0.9)	0.03
All vascular complications	18 (4.6)	13 (4.7)	5 (4.4)	1.0
Cardiac tamponade	2 (0.5)	2 (0.7)	0 (0.0)	1.0
**Postprocedural laboratory data**				
eGFR from cystatin C, ml/min/1.73 m²	48.0 (35.9–63.1)	40.6 (33.1–49.8)	71.1 (64.7–77.1)	<0.001
eGFR from creatinine, ml/min/1.73 m²	52.8 (42.0–66.0)	47.2 (37.2–55.6)	69.7 (61.9–79.0)	<0.001
BNP, pg/ml	95.5 (48.2–204.8)	120.0 (58.6–234.8)	56.6 (32.4–106.3)	<0.001
**Postprocedural echocardiographic data**				
Peak AV velocity, m/s	2.1 (1.8–2.5)	2.1 (1.8–2.5)	2.1 (1.8–2.5)	0.95
Mean AVPG, mmHg	9 (7–12)	9 (7–12)	9 (7–13)	0.89
EOA, cm²	1.56 (1.37–1.78)	1.56 (1.38–1.77)	1.58 (1.33–1.80)	0.90
Moderate or severe AR, *n* (%)	20 (5.1)	14 (5.2)	6 (5.4)	1.0
Moderate or severe MR, *n* (%)	17 (4.4)	14 (5.2)	3 (2.7)	0.40

Categorical variables are shown as numbers (percentages) and continuous variables are shown as medians (25–75th percentiles).

eGFR, estimated glomerular filtration rate; BNP, brain natriuretic peptide; AV, aortic valve; AVPG, aortic valve pressure gradient; AVA, aortic valve area; AR, aortic regurgitation; MR, mitral regurgitation; EOA, effective orifice area.

### 3.2. The 3-year prognostic impact of CKD calculated from cystatin C after TAVI

The total study population was divided into two groups (CKD or non-CKD), which was estimated by eGFR (cystatin) at the time of discharge. There were significant differences in age, STS risk score, prevalence of New York Heart Association functional class III or IV, peripheral artery disease, atrial fibrillation, and pulmonary disease between the two groups. In pre-procedural investigations, plasma albumin level, plasma natrium level, plasma hemoglobin level, plasma BNP level, and LVEF showed significant differences between the two groups. Additionally, at the time of discharge, there were significant differences in the two groups regarding the duration of the procedure, life-threatening/major bleeding, BNP, and eGFR calculated from both cystatin C and creatinine (Tables 1, 2). The total number of all-cause deaths was 46 (non-CKD group 5, CKD group 41).

**Figure 2:**
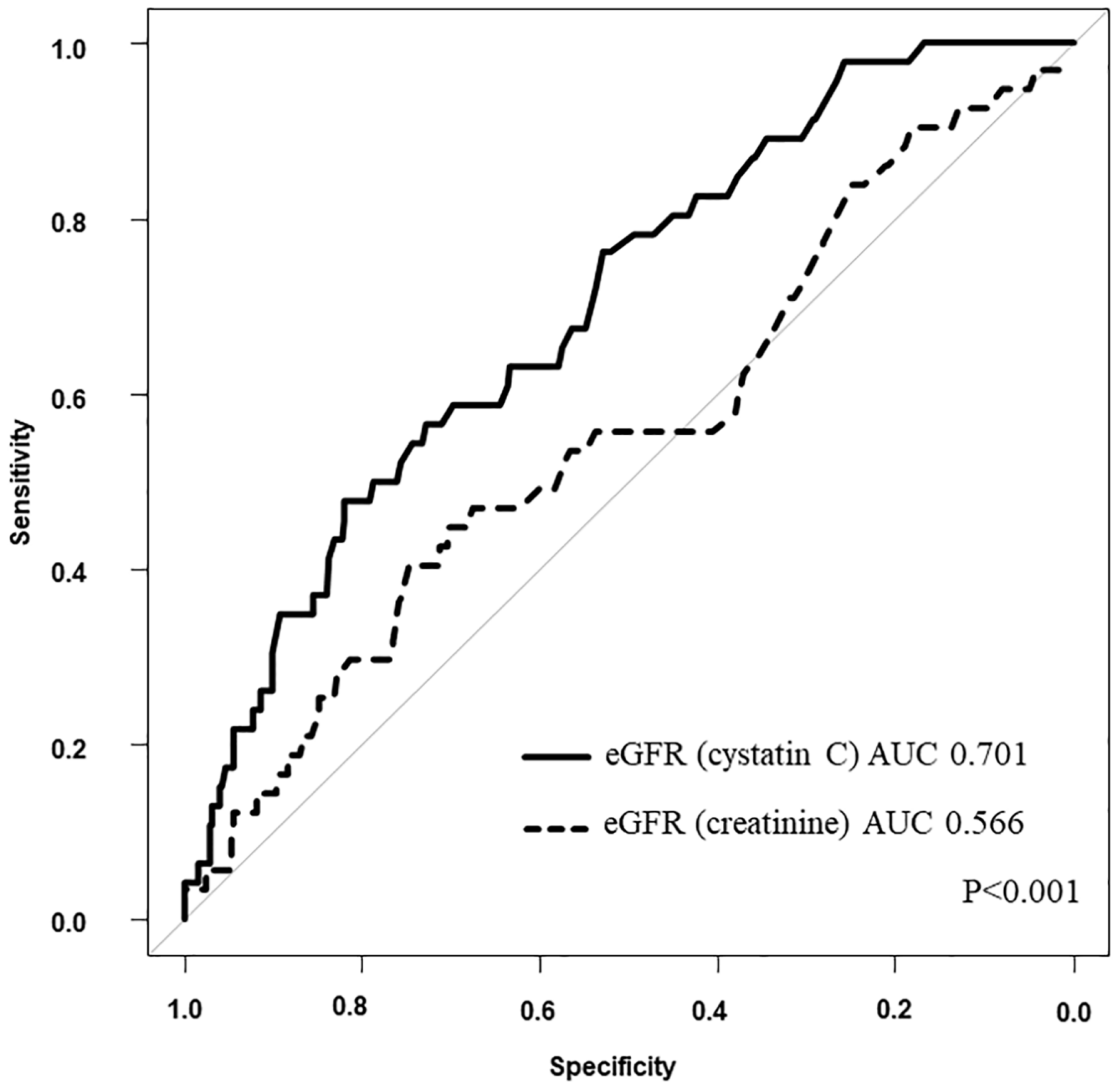
Comparison between eGFR (cystatin C) and eGFR (creatinine). AUC, area under the curve; eGFR, estimated glomerular filtration rate.

The results of the univariate Cox regression analysis for the association between cumulative mortality and clinical findings are presented in Table 3. The analysis indicated that eGFR (cystatin C), diabetes mellitus, liver disease, plasma albumin level on admission, and plasma BNP level at discharge were associated with 3-year all-cause mortality. Table 4 shows the multivariate Cox regression analysis for two models—model 1 includes eGFR (cystatin C) and model 2 includes eGFR (creatinine). In model 1, the multivariate Cox regression analysis indicated that eGFR (cystatin C) (HR, 0.972; 95% CI, 0.953–0.990; *p* = 0.003), diabetes mellitus (HR, 2.090; 95% CI, 1.149–3.811; *p* = 0.02), and liver disease (HR, 2.813; 95% CI, 1.089–7.266; *p* = 0.03) were independently associated with 3-year all-cause mortality. In contrast, model 2 showed that the 3-year all-cause mortality was not independently associated with eGFR (creatinine) (HR, 1.002; 95% CI, 0.985–1.019; *p* = 0.82); diabetes mellitus (HR, 1.954; 95% CI, 1.072–3.564; *p* = 0.03), liver disease (HR, 2.960; 95% CI, 1.142–7.764; *p* = 0.03), plasma albumin level on admission (HR, 0.398; 95% CI, 0.212–0.744; *p* = 0.004), and plasma BNP level at discharge (HR, 1.001; 95% CI, 1.000–1.001; *p* = 0.02) were independently associated. Figure 2 shows a comparison between the predictive value for the 3-year all-cause mortality for eGFR (cystatin C) and eGFR (creatinine) using a ROC curve, which demonstrated that the predictive value of eGFR (cystatin C) is significantly higher than that of eGFR (creatinine) (AUC 0.701 vs. 0.566; *p* < 0.001). In model 3, the multivariate model with age and male sex indicated that eGFR (cystatin C) (HR, 0.963; 95% CI, 0.946–0.980; *p* < 0.001), was independently associated with 3-year all-cause mortality. In contrast, model 4 showed that the 3-year all-cause mortality was not independently associated with eGFR (creatinine) (HR, 0.993; 95% CI, 0.977–1.010; *p* = 0.43); male sex (HR, 1.801; 95% CI, 1.007–3.224; *p* = 0.047) was independently associated.

**Table 3:** Cox regression univariate analysis for the association between cumulative mortality and clinical findings.

Parameter	Univariate		
	Unadjusted HR	95% CI	*p*–value
eGFR from cystatin C, ml/min/1.73 m²	0.963	0.946–0.981	<0.001
eGFR from creatinine, ml/min/1.73 m²	0.994	0.977–1.010	0.44
Age	1.001	0.944–1.060	0.98
CFS	1.371	0.964–1.950	0.08
NYHA Class III or IV	1.258	0.660–2.397	0.49
Diabetes mellitus	2.037	1.126–3.687	0.02
Hypertension	0.532	0.237–1.193	0.13
Liver disease	3.527	1.389–8.955	0.008
Pulmonary disease	1.564	0.699–3.499	0.28
Albumin on admission	0.344	0.190–0.626	<0.001
BNP at discharge	1.001	1.000–1.002	<0.001
Preprocedural LVEF	0.995	0.969–1.022	0.72
Postprocedural EOA	1.388	0.561–3.436	0.48
Hemoglobin on admission	0.884	0.735–1.065	0.19
Transfemoral	0.989	0.306–3.196	0.98

eGFR, estimated glomerular filtration rate; CFS, clinical frailty scale; NYHA, New York heart association; BNP, brain natriuretic peptide; LVEF, left ventricle ejection fraction by modified simpson methods; EOA, effective orifice area; HR, hazard ratio; CI, confidence interval.

**Table 4:** Cox regression multivariate analysis for the association between cumulative mortality and clinical findings; model 1, 2, 3 and 4.

Parameter	Model 1			Model 2		
	Adjusted HR	95% CI	*p*-value	Adjusted HR	95% CI	*p*-value
eGFR from cystatin C, ml/min/1.73 m²	0.972	0.953–0.990	0.003	−	−	−
eGFR from creatinine, ml/min/1.73 m²	−	−	−	1.002	0.985–1.019	0.82
Diabetes mellitus	2.090	1.149–3.811	0.02	1.954	1.072–3.564	0.03
Liver disease	2.813	1.089–7.266	0.03	2.960	1.142–7.674	0.03
Albumin on admission	0.509	0.258–1.005	0.051	0.398	0.212–0.744	0.004
BNP at discharge	1.001	1.000–1.001	0.10	1.001	1.000–1.001	0.02

Parameter	Model 3			Model 4		
	Adjusted HR	95% CI	*p*-value	Adjusted HR	95% CI	*p*-value
eGFR from cystatin C, ml/min/1.73 m^2^	0.963	0.946–0.980	<0.001	−	−	−
eGFR from creatinine, ml/min/1.73 m^2^	−	−	−	0.993	0.977–1.010	0.43
Age	0.985	0.929–1.045	0.62	1.002	0.944–1.063	0.96
Male	1.732	0.960–3.123	0.68	1.801	1.007–3.224	0.047

eGFR, estimated glomerular filtration rate; BNP, brain natriuretic peptide; HR, hazard ratio; CI, confidence interval.

**Figure 3:**
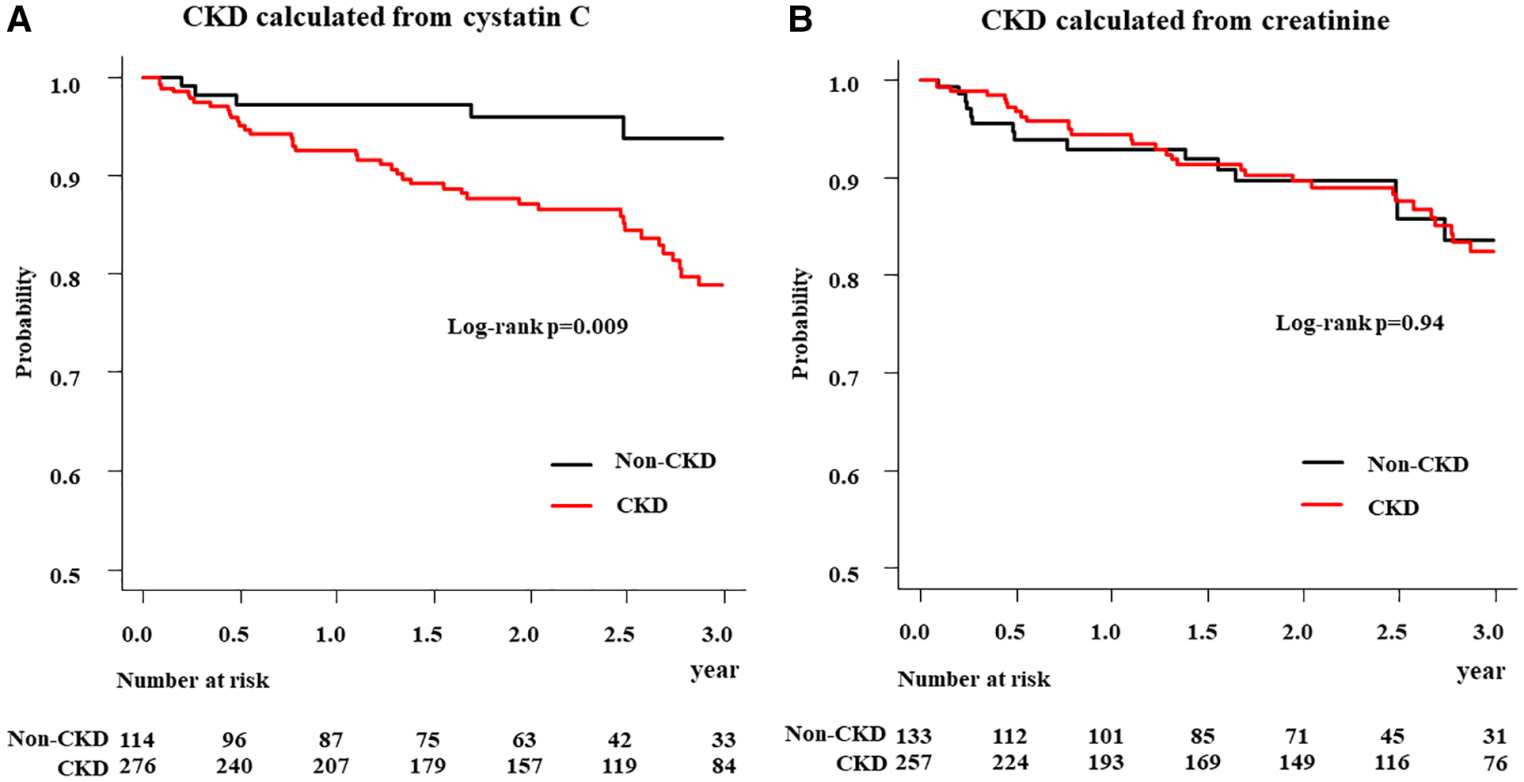
Kaplan–meier analysis of all-cause mortality in patients in CKD and non-CKD groups. (**A**) CKD calculated from cystatin C (**B**) CKD calculated from creatinine; CKD, chronic kidney disease.

Kaplan–Meier estimates revealed that 3-year all-cause mortality were higher in the CKD (cystatin C) group than that in the non-CKD (cystatin C) group with log-rank *p* = 0.009. In contrast, there was no significant difference between the CKD (creatinine) and non-CKD (creatinine) groups with log-rank *p* = 0.94 (Figure 3).

## 4. Discussion

In this study, we demonstrated that CKD estimated by cystatin C (not by creatinine), predicted the 3-year all-cause mortality in patients undergoing TAVI. Our study showed that eGFR (cystatin C), diabetes mellitus, and liver disease were independently associated with 3-year all-cause mortality in the TAVI cohort. In addition, eGFR (cystatin C) presented good calibration, and better discrimination than eGFR calculated from serum creatinine. Furthermore, the estimated 3-year mortality rate was significantly higher in the CKD group, for which eGFR was calculated from cystatin C, as compared to that in the CKD group where eGFR was calculated from serum creatinine. To the best of our knowledge, this is the first study to demonstrate that CKD (calculated from cystatin C-based eGFR) is associated with long-term mortality in the TAVI cohort.

### 4.1. Comparison of the 3-year prognostic impact of CKD calculated from cystatin C and serum creatinine

CKD is a major risk factor for death and disability following TAVI, and is common in patients undergoing TAVI. Multiple studies suggest that approximately 91% patients have stage ≥ 2 CKD (7–9, 22–24). eGFR (creatinine) is generally used to assess renal function in clinical settings; however, it is well known that the non-GFR determinants of serum creatinine, including muscle mass, diet, and physical activity can confound the associations between creatinine-based eGFR and outcomes (25). The TAVI cohort is predominantly older; therefore, using creatinine in this cohort to calculate eGFR may lead to an overestimation of the GFR. However, serum cystatin C is not a blood test measurement used in all clinical settings, but it is unaffected by age, gender, or muscle mass; thus, renal function calculated from serum cystatin C can be more accurately evaluated in TAVI patients (11–15). Consistent with these findings, we demonstrated that CKD estimated by cystatin C (and not creatinine) predicted the 3-year all-cause mortality in patients undergoing TAVI. It was previously reported that assessment of CKD with serum cystatin C did not improve mortality prediction compared to serum creatinine in older community dwelling British men (median age 78.4 years) (26). In addition, although previous studies have reported that CKD assessed by creatinine is a poor prognostic factor following TAVI (7–9), eGFR (creatinine) was not shown to be associated with 3-year all-cause mortality following TAVI in this study. We speculate that this discrepancy is due to patient characteristics. Our study cohort included many older patients (median age 84.0 years) and a significant number of women, with the percentage of men being 32.8%, moreover a smaller body size (median BSA 1.43 m^2^). These characteristics significantly affect body composition and frailty and serum creatinine may underestimate renal function. In conclusion, we believe that eGFR (cystatin C) is useful for predicting mortality following TAVI, and that it is superior to eGFR (creatinine), particularly for the older cohort, which includes many frail and undernourished patients.

### 4.2. Potential clinical implications

In this study, serum creatinine and cystatin C at discharge following TAVI were used for evaluation. Cubeddu RJ, et al. reported that in patients with severe aortic stenosis undergoing TAVI, renal function is more likely to stay the same or improve than worsen (24). They also reported that renal function following TAVI is associated with all-cause mortality at one year (24). Mizutani K, et al. reported that BNP at discharge would be more favorable for risk stratification of long-term prognosis than that on admission in patients who had undergone TAVI because the TAVI procedure releases the left ventricle from pressure overload immediately after the valve replacement (27). We thought renal function at discharge, as well as BNP, to be more useful in assessing its relationship to prognosis. Therefore, we considered renal function following TAVI to be suitable for assessing the relationship to prognosis. The prevalence of postoperative AKI was low (4.1% of the total) and did not appear to influence the prognostic analysis. Serum cystatin C also has a greater diagnostic sensitivity than that of serum creatinine, especially in the pre-CKD area of serum creatinine (eGFR 75 ml/min/1.73 m^2^ to 88 ml/min/1.73 m^2^) (28). These characteristics may affect the predictive value of long-term mortality in patients with cardiovascular disease. Additionally, it has been reported that cystatin C is a predictor of long-term prognosis and rehospitalization in patients with heart failure and percutaneous coronary intervention (29–31). With the expanded indication of TAVI for a subset of lower-risk patients, TAVI could be considered as the first-line treatment choice for patients with AS. This would show improvement in pre-procedural, short-term, and long-term endpoints. Cystatin C could be easily monitored with blood tests. Accordingly, CKD patients should be followed-up carefully.

### 4.3. Study limitations

This study has several limitations. First, this study was a single-center design, and the small study population (*n* = 390) for the time period reduced the statistical power of the study. Second, the median follow-up period was 808 (368–1145) days, with some cases having a shorter follow-up period. Third, the patients whose data regarding the cystatin C level at discharge were not available were excluded, which could have led to a selection bias. Fourth, cystatin C is not a test commonly measured in clinical practice and has limited use.

### 4.4. Conclusion

eGFR calculated from cystatin C at the time of discharge was associated with the 3-year all-cause mortality in patients undergoing TAVI. Thus, serum cystatin C is superior to serum creatinine as a prognostic biomarker for the TAVI cohort.

## Data Availability

The raw data supporting the conclusions of this article will be made available by the authors, without undue reservation.

## Author's note

This study used the same TAVI study population as in the “Kihon checklist is useful for predicting outcomes in patients undergoing transcatheter aortic valve implantation” (32) reported previously by Kure Y, Okai T, et al. The period covered in this study was from January 2016 to December 2021, a different time period than in paper (32). Paper (32) included a cohort of TAVI patients who had undergone TAVI at Osaka Metropolitan University Hospital and were able to be evaluated for frailty, while the current study includes all TAVI patients who underwent TAVI at Osaka Metropolitan University Hospital. (peri-complicated in-hospital deaths, patients with active cancer, and cystatin C deficiency at discharge were excluded).

The TAVI procedure was the same as in paper (32), and the TAVI procedrure at Osaka Metropolitan University Hospital has been consistently the same in the present study.

The same analysis methods and software were used as in paper (32). The present study is a two-group comparison, whereas paper (32) is a three-group comparison.

Paper (32) investigated the impact of frailty on prognosis in TAVI patients, and reported the effectiveness of the Kihon checklist as one of the evaluation methods. The current study investigated the relationship between renal function calculated from cystatin C and prognosis after TAVI. From the above points, this study is different from the paper (32).
